# Altering the coffee-ring effect by adding a surfactant-like viscous polymer solution

**DOI:** 10.1038/s41598-017-00497-x

**Published:** 2017-03-29

**Authors:** Changdeok Seo, Daeho Jang, Jongjin Chae, Sehyun Shin

**Affiliations:** School of Mechanical Engineering, Korea University, Seoul, 136-701 Republic of Korea

## Abstract

A uniform deposition of the suspended particles in an evaporating droplet is necessary in many research fields. Such deposition is difficult to achieve, because the coffee-ring effect dominates the internal flow in a droplet. The present study adopts a biocompatible, surfactant-like polymer (Polyethylene glycol, PEG) to break the coffee-ring effect and obtain a relatively uniform deposition of the microparticles with yielding multi-ring pattern over a droplet area. Movements of the suspended particles in evaporating droplets and deposition patterns of them on a glass substrate were analyzed with microscopic images and video files. The PEG in the droplets successfully altered the coffee-ring effect because of the surface tension variation, which induced a centripetal Marangoni flow. Balancing these two phenomena apparently generated the Marangoni vortex. For PEG solution droplets, the pinning–depinning process during evaporation was periodically repeated and multiple rings were regularly formed. In conclusion, adding a surfactant-like viscous polymer in a droplet could provide a uniform coating of suspended particles, such as cells and various biomaterials, which would be essentially required for droplet assays of biomedical applications.

## Introduction

The evaporation of a sessile droplet, which is by itself an interesting encounter in everyday life, causes a complex, non-equilibrium fluid dynamic phenomenon^1^. The fluid dynamic complexity of a drop is caused by the non-uniform evaporating flux over its gas–liquid interface and the thermal gradient–induced Marangoni stress^2^. The liquid within the droplet will flow outward the edge when the Marangoni effect is negligible to compensate the large evaporation loss of liquid at the edge^3^. Subsequently, the suspended particles are driven to the edge and deposited in a ring-like pattern, known as the coffee-ring effect^1, 2, 4^. The coffee-ring phenomenon occurs when the droplet is pinned at its contact line^1, 2, 4^. Occasional depinnings of the contact line may result in concentric rims on the solid surface^5^. Poly-disperse particles are orderly deposited from the edge to the center in a size-dependent manner^6^. The coffee-ring effect is observed for various particle sizes ranging from microparticles to nanoparticles^6, 7^, molecules^5^, and ions^5^.

However, the coffee-ring stain phenomenon is a crucial defect in fields of inkjet printing^8^, microdot array^9^, and other coating technologies that need uniform depositions. Uniform coating is also important for the sensing and detection assay in biotechnology. Probe materials, such as antibodies for virus detection, are commonly coated on a substrate as a droplet pattern. However, most probe materials are highly concentrated near an edge, and the surface contact area is highly restricted because of the coffee-ring effect. The probe sensitivity would then significantly decrease. Therefore, many research groups are making efforts to obtain a uniform deposition by suppressing the coffee-ring effect. A typical example is the Yunker *et al*.’s report^10^ which demonstrated interaction between anisotropic particles suppresses the coffee-ring effect and lead to a uniform deposition of particles. This report was uniquely done without any chemical manipulation.

In fact, there have been many notable reports to overcome coffee-ring effect with controlling liquid properties such viscosity and pH and altering temperatures of substrate and droplet. Cui *et al*.^11^ demonstrated when an added hydrosoluble polymer increased the solution viscosity, resulting in a large resistance to the radially outward flow and subsequently a small amount of spheres deposited at droplet edge. Also, Bhardwaj *et al*.^12^ demonstrated the effect of the pH of the solution on the deposit pattern, which is the results of force interactions between substrate and particles such as the electrostatic and van der Waals forces. Parsa *et al*.^13^ reported that depending on the substrate temperature, three distinctive deposition patterns are observed: a nearly uniform coverage pattern, a “dual-ring” pattern, and multiple rings corresponding to “stick–slip” pattern. Hu *et al*.^2, 14, 15^ utilized the temperature-dependent surface tension of liquid and reported a repulsive Marangoni force in the evaporating droplet by the latent evaporation heat.

Meanwhile, Still *et al*.^16^ visualized the surfactant-driven Marangoni effect using suspended microparticles. A small ionic surfactant (i.e., Sodium dodecyl sulfate, SDS) induced the Marangoni flow because of a surface tension gradient at a droplet’s air–water interface. The result of the evaporation induced outward flow and the inward Marangoni flow is the “Marangoni vortex (eddy)” phenomenon^16^. This phenomenon induces a uniform deposition of the evaporating droplet by forming tree-like multiple rings. Kajiya *et al*.^17^ developed a method to determine the concentration profiles in the evaporating droplets of polymer solution by combining the fluorescence intensity measurement and the lateral thickness profile measurement of a droplet. This experimental protocol was exactly adopted in the present study to determine polymer concentration profile in a droplet. Recently, Kim *et al*.^18^ reported that multiple sequential Marangoni flows and particle-surface interactions are key parameters to achieve uniform particle coatings in a droplet and suggested a small concentration of surfactant and surface-adsorbed polymer via physisorption for a uniform deposit in a binary mixture.

In this research, we made an effort to deposit suspended particles more uniformly by adding polyethylene glycol (PEG). PEG is known as a non-toxic and non-immunogenic polymer widely used in food, cosmetic, and pharmaceutical research fields^19, 20^. Various molecular weights and functionally modified PEGs are available for use. It is worthy to note that PEG molecules in water, which are solution-like surfactants^21, 22^, cause to decrease surface tension and to increase the solution viscosity in a concentration-dependent manner^23, 24^. Also, adding PEG would induce Marangoni flow, resulting in change of de-pinning characteristics of contact line. We added a biocompatible surfactant polymer in a droplet solution and expected to obtain a better homogenous coating of suspended microparticles because of the combined effects of surface tension and viscosity changes in the evaporation process.

Therefore, the present study is designed to understand the existing mechanisms of the internal flow and coating pattern in a PEG solution droplet. The internal flow patterns were carefully analyzed by observing the motions of suspended particles. The local PEG concentration profile in an evaporating droplet was also carefully measured through the fluorescence microscopy according to the proven experimental method^17^. The corresponding surface tension and viscosity profiles were subsequently analyzed after the calibration process. Furthermore, we examined and compared the evaporating characteristics of four different fluids (i.e., water, purely viscous fluid and SDS- and PEG-added solutions) to delineate the combined effects of surface tension and viscosity on a droplet’s internal flow. The purely viscous fluid was prepared using a polyvinylpyrrolidone (PVP), which is a well-known aqueous polymer that increases fluid viscosity^25, 26^ and does not change the surface tension. SDS is an aqueous ionic surfactant that decreases the surface tension of the air–water interface and does not change the solution viscosity^16^.

## Result

### Deposition patterns

**Figure 1:**
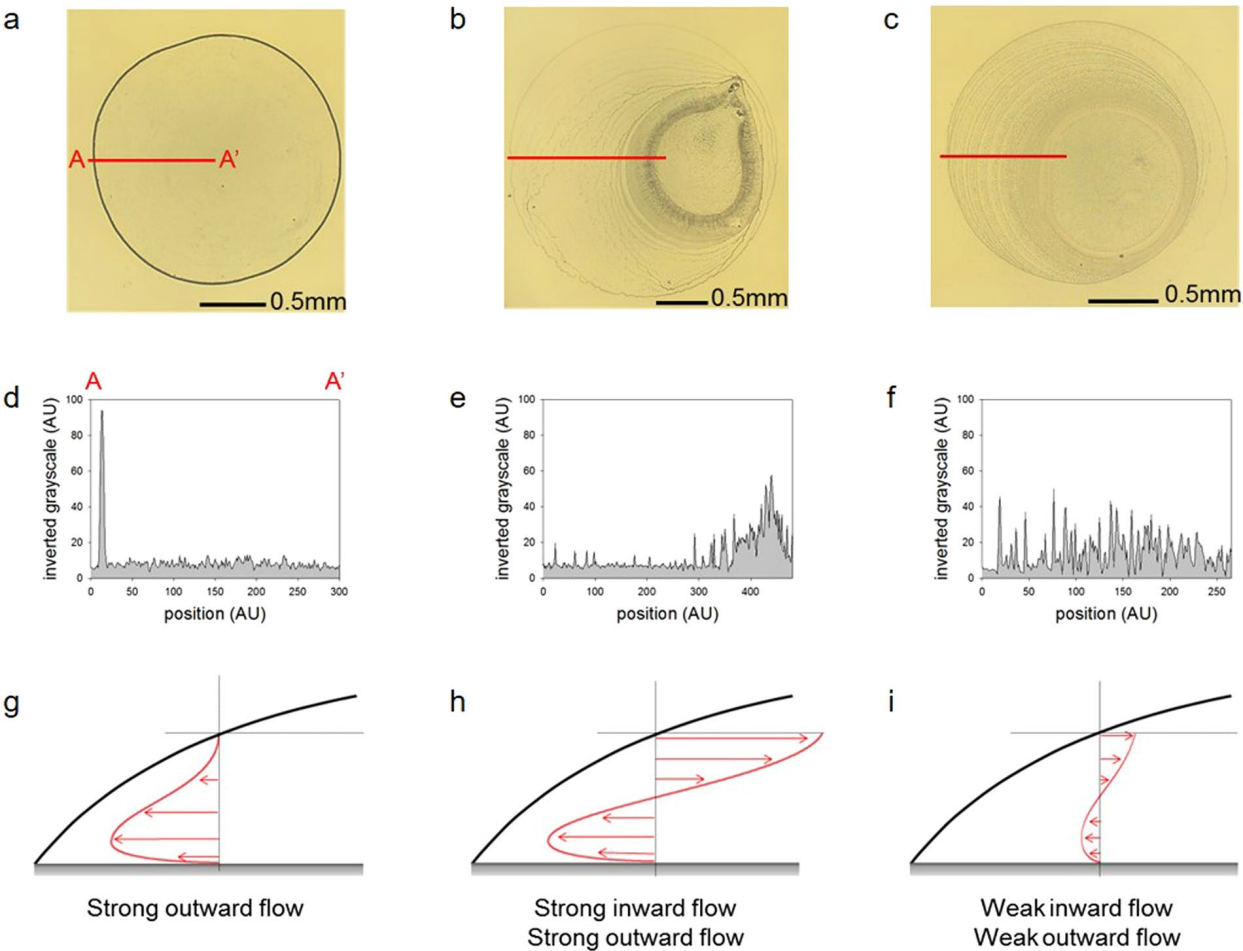
Deposition patterns of the droplets and schematic image of fluidic motion in the evaporating droplets. Magnified bottom views of the deposition patterns of droplets of the (**a**) DIw and (**b**) SDS and (**c**) PEG solutions. Profiles of the inverted greyscale intensity of the (**d**) DIw and (**e**) SDS and (**f**) PEG solutions. Schematic cartoons of the fluidic motions in the evaporating droplets of the (**g**) DIw and (**h**) SDS and (**i**) PEG solutions.

The standard coffee-ring effect appears in the deposition of suspended polystyrene (PS) particles dispersed in a distilled water (DIw) droplet (Fig. 1a). The SDS effectively reduces the coffee-ring effect, but generates another problem (i.e., the particles focus on the central area) (Fig. 1b). The deposition of the particles is more uniform when PEG is incorporated to the liquid as can be seen in Fig. 1c. These three images are quantitatively analyzed by performing an intensity analysis (Fig. 1d–f), as described in the Methods section. The greyscale intensity may indicate the coated depth through microparticles^16^. Overall, the PEG droplets yield a much more homogeneous pattern (Fig. 1f) than the SDS droplets (Fig. 1e). The intensity of the SDS droplet gradually increases toward the droplet center, which may be caused by the strong Marangoni flow carrying most of the particles toward the center. The PS particles in the DIw droplet are strongly and quickly driven and collected near the contact line by the outward flow (Fig. 1g). Those in the PEG droplet have a radial inward and outward motion like those in the SDS droplet (Fig. 1h). However, the motions are slower than those of the DIw and SDS droplets (Fig. 1i). These particle motions are shown well in Supplementary video 1.

### Mechanism of Marangoni vortices

**Figure 2:**
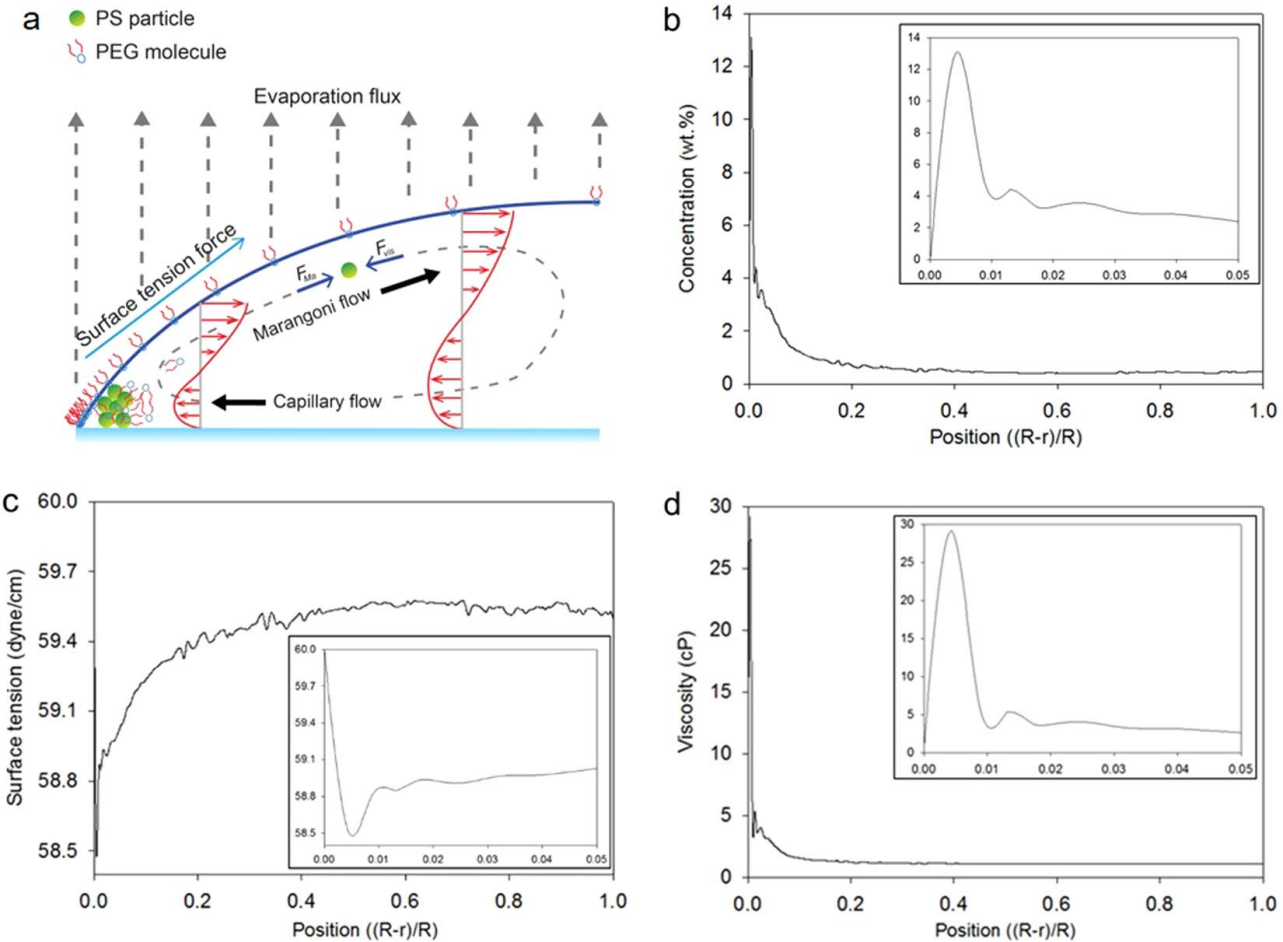
Mechanism of the PEG-induced Marangoni vortex. (**a**) Schematic image of the suspended particles and fluidic motion in the evaporating droplet system. (**b**) A profile of the PEG concentration by position. (**c**) A profile of the surface tension. (**d**) A profile of the viscosity. The expanded profiles near the edge are shown as the insets of each figure.

Radial outward flow, which is commonly observed in coffee-ring phenomena, is induced by non-uniform evaporation rate in radial direction. As shown in Fig. 1(g), particles tend to move outward and are accumulated near a contact line, forming coffee-ring effect. Generally, the outward flow is strongly observed near the bottom surface. Meanwhile, as depicted in Fig. 1(h) and (i), the suspended particles in SDS and PEG solutions shows radial inward flow as well as radial outward flow. The inward flow, so called Marangoni flow, was induced by a surface tension gradient. The surface tension gradient is maximized along the interfacial surface between air and liquid and thus the inward flow would be formed near the air-liquid interfacial line. When the magnitude of Marangoni flow is comparable to the capillary flow, then these two opposite-directional flows are connected and formed a closed-loop flow. The flow rotation is so called “Marangoni vortex” or “Marangoni eddy”. Figure 2(a) depicts the schematic image of the Marangoni vortex near the contact line. The mechanism of the vortex can be delineated with examining the local properties of liquid in a droplet.

The local concentration profiles were determined using fluorescent-intensity measurement method, which can be found elsewhere^17^. Surface tension of PEG solution was experimentally measured with increasing PEG concentration, as shown in the Supplementary Fig. 2a. Then, we obtained a relationship between the PEG concentration and the surface tension of the PEG solution, which can convert local concentration profiles to local surface tension ones. Similarly, viscosity profile was also obtained with measurement of viscosity of PEG solution vs. PEG-concentration (Supplementary Fig. 2b). The PEG concentration yielded a peak at about 3 μm apart from the contact line and sharply decreased near a contact line, as shown in Fig. 2b. Similar results were also reported in the previous study^17^ but the mechanism was fully elucidated.

Figure 2(b) describes the concentration profile of the PEG along the radial direction from the edge to the center in a droplet. The concentration profile is depicted when a particle begins to move inward at t = 10 s (*t*/*t*_e_ = 0.036). The initial PEG droplet concentration was 0.5 wt.%, which is the same as the bulk concentration near the center (i.e., (R-r)/R = 1.0), as shown Fig. 2(b). The total time to complete evaporation (*t*_e_) is about 280 s. PEG polymers were rapidly accumulated and became 13.1 wt.% near the edge because of the outward flow associated with the coffee-ring effect. Consequently, the surface tension significantly decreased and yielded the maximum gradient of surface tension near the edge. Also, the bulk viscosity of the PEG droplet was 1.08 cP, while the edge viscosity was 29.15 cP.

### Pinning and depinning process

**Figure 3:**
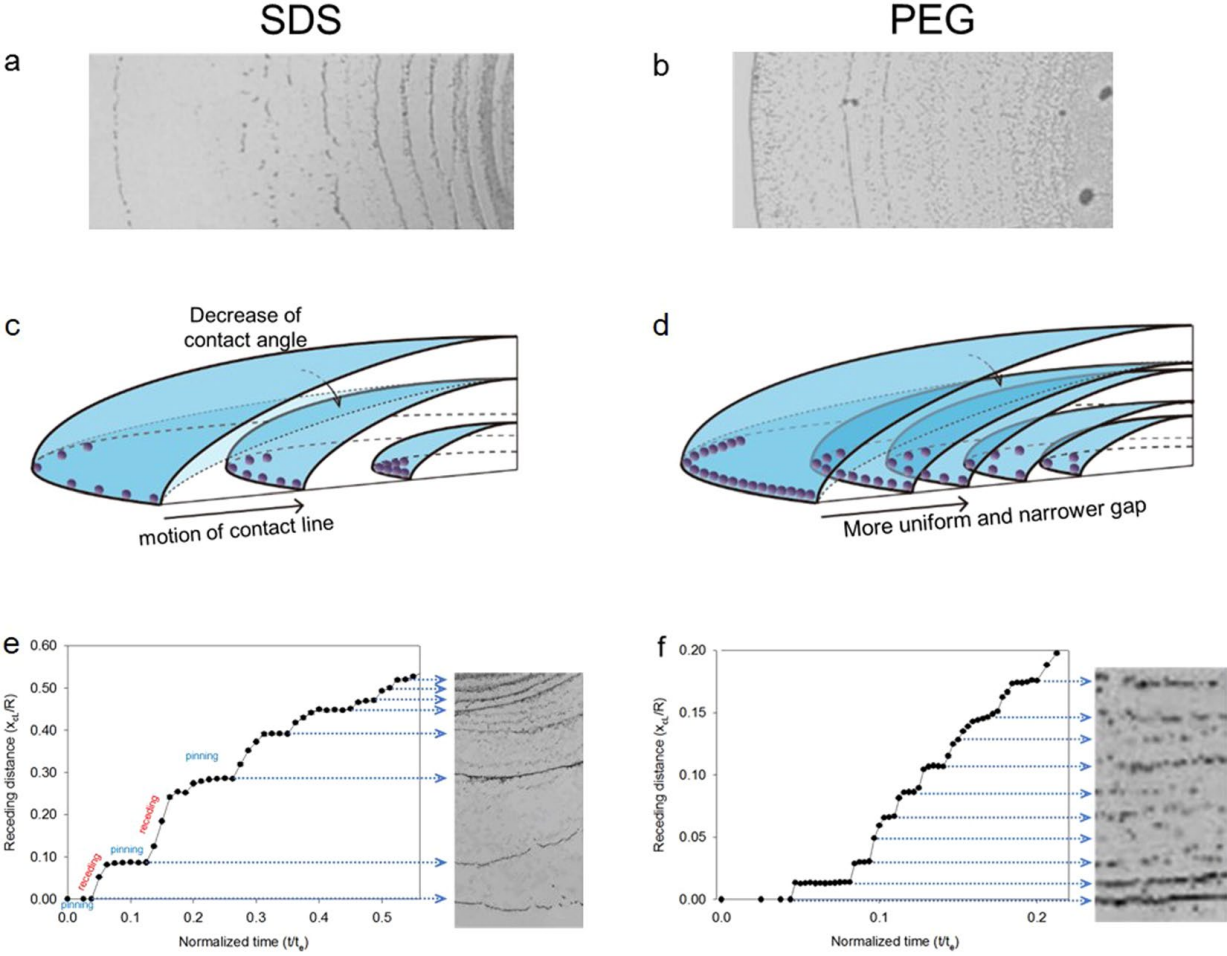
Analyses of the contact line motion: pinning and receding. Photographic images depicting the multiple rings observed in the (**a**) SDS and (**b**) PEG droplet. Schematic images of the (**c**) SDS and (**d**) PEG droplet during evaporation. Profiles of the contact line migration distance (*x*_*c*_) of the (**e**) SDS and (**f**) PEG droplets from the initial contact line by time. The distance and length were measured five times.

**Figure 4:**
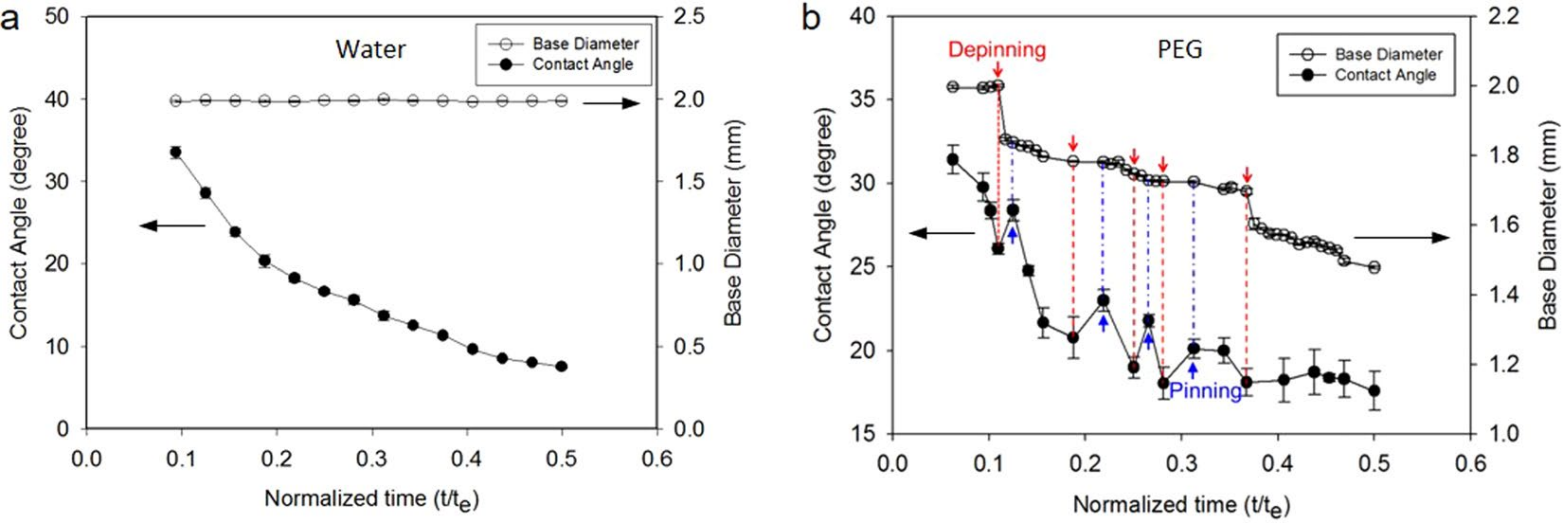
Variation of contact angle during pinning and receding. (**a**) water and (**b**) PEG.

Figure 3 shows the analyses of the contact line motion for SDS and PEG solutions. Both SDS and PEG solutions formed multiple-ring patterns as shown in Fig. 3(a) and (b). These multiple rings were a result of the repeated pinning and receding process. Comparing these multiple rings for SDS and PEG, one can easily distinguish that SDS shows relatively larger gap between rings and fewer number of rings than PEG does. These findings can be confirmed with analyzing the migration distance of the contact line (*x*_*cL*_/*R*) with respect to time in Fig. 3(e) and (f). For the SDS droplet, a high stair pattern can be clearly observed in the pinning and receding process. The height of the stair, which was a migration length per depinning event, ranged from 150 to 400 μm. In particular, the first three receding distances of the contact line for SDS were much larger than those for PEG. In particular, the accumulated distance of the first three receding processes for SDS is 0.4 (*x*_*cL*_/*R*), which covers 64% of area in a droplet. Meanwhile, the pinning and depinning process for the PEG droplet was similar to the low stair pattern with a small-scale height (20–50 μm). For PEG, the total distance during first three receding processes is 0.05, which is quite small. In fact, the number of the rings for SDS and PEG is 8 and 10, respectively and there is not significant difference. Thus, the initial receding distances (or step heights) would determine the uniformity of particle deposition. The re-pinning from receding process would be affected by contact angle recovery associated surface tension^27^ and adhesive force between solute and solid surface^18^. The variation of contact angle during pinning-and receding processes was analyzed in Fig. 4.

### Contact angle variation during pinning and receding

Figure 4 shows the variation of the contact angle (CA) and base diameter (BD) over time during evaporation. For water, since the contact line is pinned, the CA deceases with time and the BD remains constant. Meanwhile, the PEG solution yields pinning and receding processes. For initial pinning duration, the CA deceases from 31.4° while and the BD is constant, as shown in Fig. 4(a). The first receding occurs at CA = 26.1°, which is determined as the receding contact angle (*θ*_*r*_). Contrast to the first receding CA, the second and the third ones are relatively small and range between 18.1° and 20.8°. After the fifth depinning, the CA slightly fluctuates while the BD decreases monotonically. It is worthy to note that for receding process, the contact angle rapidly increases, as shown Fig. 4(b). Similar results were reported in a previous study^28^. However, Li *et al*.^28^ reported that the CA was kept constant during receding process. The different results of contact angle hysteresis might be due to the surface conditions including hydrophobicity, surface electrical potential and particle concentration^29^.

### Characteristics of Marangoni vortices

**Figure 5:**
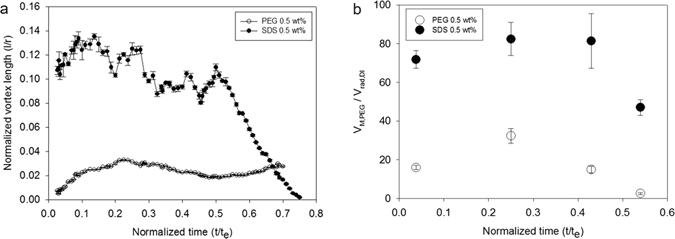
Characterization of Marangoni vortex. (**a**) Comparison of Marangoni vortex between SDS and PEG droplet, (**b**) Variation of Marangoni vortex velocities for SDS and PEG droplet.

The time-variant changes of the Marangoni vortices are analyzed in Fig. 5. Five particles are traced, and vortex lengths are measured via video analysis for the quantitative analysis. The average length value is plotted as a function of time in Fig. 5a. The vortex for the SDS droplet (closed circle) grows quickly, and its length reaches maximum at 160 μm (*l*/*R* = 0.16) at *t*/*t*_*e*_ = 0.1. The vortex length of SDS fluctuates with time and rapidly decreases after *t*/*t*_*e*_ = 0.5. Meanwhile, for a PEG droplet, the vortex was initially quite small (~7 μm) gradually increases with time up to 33 μm (*l*/*R* = 0.033) at *t*/*t*_*e*_ = 0.23. Then, the vortex disappears at *t*/*t*_*e*_ = 0.7. Meanwhile, the particle velocities of SDS are much faster than those of PEG, as shown in Fig. 5b. For scale analysis, the Marangoni velocity (*V*_*M*_) was normalized with the average velocity of radial outward flow for water (*V*_*rad*,*w*_), which is 4.8 μm/s. At *t*/*t*_*e*_ = 0.43, a typical Marangoni velocity of SDS is 390 μm/s, which is 82 times of V_rad,w_, whereas that of PEG is 72 μm/s, which is 17 times of *V*_*rad*,*w*_. Interestingly, the suspended particles in the SDS and PEG droplet begin to move at different points of time during the early stage of the evaporation process. When a droplet is posted on a substrate, the Marangoni flow immediately occurs in SDS. However, it takes for 10 seconds for PEG droplet (0.5%) to observe the inward Marangoni flow. Since the PEG concentration profile varies with time as shown in Supplementary Fig. 1(e), it would take a time to build up a potential (surface tension gradient) to generate the Marangoni flow. Thus, when the Marangoni flow begins, it may be called as an onset (or critical) condition of Marangoni phenomena, which will be discussed later.

## Discussion

As explained in the introduction, various factors affect deposition pattern of microparticles in a droplet. It is known that the deposition pattern can be altered by change of surface activity through pH control^12^, temperature-dependent surface tension gradient^2, 13–15^, surfactant-induced surface tension gradient^16–18^, surface-mediated repulsion of different shaped particles^10^, and etc. Even though it is a common and simple evaporation phenomenon of a particle-suspended droplet, it includes various physics and chemistry-related mechanisms and is highly complex to understand the phenomenon considering multiple affecting factors. Thus, we focused on the effect of aqueous polymer surfactant on the behavior of microparticles in a droplet. With adding the PEG in water, we could alter surface tension and viscosity of droplet, which induce not only frequent stick-and-slip motion but also a uniform multi-rings deposition pattern.

The standard deviation was adopted as a criterion to fairly estimate the uniformity of the deposition patterns of the tested samples. The effective area deposited with particles was selected from the deposition images (Fig. 1a–c). The standard deviation of the intensities on the effective area was also calculated. The PEG droplet had the lowest standard deviation value (9.34) when compared to the DIw (14.61) and SDS (12.44) droplets. This result indicated that the PEG droplet formed the most uniform deposition pattern with multiple rings among the tested samples. This method considered a completely effective area. Hence, the uniformity was more fairly estimated compared to the previous methods^10, 11, 16^.

The suspended particles in the SDS droplet begin to follow the vortex flow immediately after the droplet is on a substrate, whereas those in the PEG droplet undergo only outward motion until 10 s (Supplementary video 1). In fact, one can define a critical condition when a particle begins to move inward following the Marangoni flow. The Marangoni flow has been analyzed with the Marangoni number, which is the ratio of surface tension force to viscous force $(Ma=\frac{d{\rm{\sigma }}}{dT}\frac{l{\rm{\Delta }}T}{\mu \alpha })$. However, when there were different sizes of particles (0.5~5 μm) dispersed in a droplet of either SDS or PEG solution, Marangoni vortices were observed only in small particles (<3 μm). Furthermore, the particle velocities were inversely proportional to particle size, even though the Marangoni number is identical for all particles (Supplementary video 2). Thus, the Marangoni number failed to interpret the effect of particle size on the Marangoni flow.

Considering the force balance around a particle, as shown in Fig. 2(a) in Marangoni vortex, one can suggest the Reynolds number (*Re* = *ρVD*/*μ*), which is defined as the ratio of the inertia to viscous force. However, since the inertia (*F*_*i*_~*ρVD* ^*2*^) is not the driving force, but the resultant force, the size-dependent particle velocity cannot be directly interpreted with *Re*. If we replaced the inertia with surface tension force $({{\rm{F}}}_{{\rm{s}}}={\rm{\Delta }}{\rm{\sigma }}l)$, then the Reynolds number became a modified Marangoni number (*Ma*’ = $\frac{{\rm{\Delta }}{\rm{\sigma }}l}{6\pi \mu DV}$). Here the viscous (drag) force for a particle is *F*_*D*_ = 6πμD. It is of note that both the viscosity (*μ*) and the surface tension (Δσ) are functions of time with evaporation. When the suspended 1 μm particle began to move (at t = 10 s, v = 76 μm/s) along the Marangoni flow, the Ma’ number was 4,446. We observed different phenomena with varying particle sizes. For example, a 2 μm particle began to move at t = 58 s, and the velocity was 21 μm/s. The modified Marangoni number at this moment was about 4,200, which was similar to the case with the critical number of 1 μm. Therefore, the modified Marangoni number would be helpful to interpret the onset of Marangoni flow for various sized particles in a droplet.

The migration length of the contact line per depinning event and the number of rings were influenced by the surface tension, viscosity, and other factors^27, 28, 30^. The higher surface tension of the PEG droplet might lead to the smaller slip distance between two rings. Furthermore, we found from the measured surface tension and slip distance that a lesser potential energy barrier for depinning than that in the SDS droplet was needed in the PEG droplet^27^. The higher local viscosity at the edge region of the PEG droplet yielded a higher number of rings^30^. Therefore, the PEG droplet formed denser rings than the SDS droplet (Fig. 3).

Various additional experiments were also conducted (Supplementary Fig. 3). For instance, we tried to make a droplet with increasing viscosity and reducing surface tension by adding either SDS and PVP^25, 26^ or glycerol and SDS. Either glycerol or PVP was used for increasing viscosity to match with the PEG solution. The particle motions became milder than those in the pure SDS droplet. However, unexpected particle motions and unstable contact lines were observed. The deposition patterns of these droplets were less uniform than those of the PEG droplet. The systematic feasibility tests showed that we could not find any other fluids to produce a uniform pattern as much as the PEG did.

In summary, we compared herein the deposition patterns after droplet evaporation and the movements of the suspended particles in the droplet evaporation of pure water and SDS (surfactant) and PEG (surfactant like polymer) solutions on a glass substrate. Adding either SDS or PEG to a droplet significantly altered the coffee-ring effect and formed multiple ring patterns. However, the PEG droplet yielded the most uniform multiple ring patterns of microparticle deposition among the tested solutions. The uniform multiple ring pattern in PEG droplet was mainly attributed to the characteristics of the surfactant-like polymer of PEG. The non-uniform profiles of the surface tension and the viscosity during evaporation caused a stable Marangoni flow, which significantly altered the coffee-ring effect. Also, addition of PEG caused to change the characteristics of the de-pinning and receding, which controlled the motion of contact line. The force to require depinning was seemed to be small due to PEG addition and thus easy depinning and small distance during receding were observed and consequently uniform multiple ring patterns were formed in the PEG droplet. In conclusion, PEG, which is known as a biocompatible polymer, can be utilized as a surfactant to suppress the coffee-ring effect and generate a uniform deposition for biomaterials through biomedical assays and bioprocesses.

In Fig. 5, Marangoni vortices for PEG and SDS disappeared at 0.75 and 0.71, respectively, in the normalized time scale (*t*/*t*_*e*_). However, the real life time of Marangoni vortex of PEG (298 s) was much longer than that of SDS (205 s). In fact, there was a significant difference of evaporation time (*t*_*e*_) between PEG and SDS. For instance, average evaporation time (*t*_*e*_) for PEG was 419 s, whereas that for SDS was 273 s. The difference in the evaporation time might be associated with contact angle and air-to-liquid interfacial area. In the present study, the surface tension of SDS (37.0 dyne/cm) was smaller than that of PEG (59.5 dyne/cm) at bulk measurements. Thus, the initial contact angle of SDS was smaller than that of PEG and the air-to-liquid interfacial area for SDS were larger than that for PEG. We confirmed that the initial base diameter of PEG was 2 mm and that for SDS was 3.5 mm.

Although we suggested a novel method to form a uniform multiple ring deposition pattern in this research, the tested solutions were restricted to a particular molecular weight of polymer (i.e., 35,000), polymer concentration (0.1–5.0 wt%) and particle concentration (0.5%, v/v). Even though we did not include the result of PEO solution, we observed similar Marangoni vortex for PEO, which was also observed in a previous study^31^. In fact, PEO has the same chemical structure as PEG but relatively large molecular weight (MW = 600,000, Sigma-Aldrich, USA). Since the particles were used for flow visualization, the concentration of particles was not examined. As long as there is neither severe particle interaction nor aggregation, any neutrally buoyant particles can be utilized for flow visualization. Interestingly, when the polymer concentration was higher or equal to 5 wt%, the dissolved polymer formed a mountain-like structure, as shown in Supplementary Fig. 4. Similar results were reported in a previous study^31^, which interpreted the multiple effects of high viscosity of droplet liquid, contact line friction and diffusion. Further experiments are required to fully validate these various factors, in particular with regards the particle size and concentration.

## Materials and Methods

The polyethylene glycol (PEG), polyvinylpyrrolidone (PVP), and sodium dodecyl sulfate (SDS) powders were purchased from Sigma-Aldrich, Korea (www.sigmaaldrich.com). The molecular weights of PEG and PVP were 35,000 and 40,000, respectively. The polystyrene (PS) microparticle and fluorescence microparticle suspensions were purchased from ThermoFisher Scientific (MA, USA). The PS microparticle diameter was 1.019 μm. The fluorescence polystyrene (FPS) microparticle had a diameter of 0.025 μm. The excitation and emission wavelengths were 468 nm and 508 nm, respectively. Both solids had an initial concentration of 1% (v/v), while the particle densities were 1.05 g/cm^3^. The microscope slide glasses made from soda lime glasses were purchased from Marienfeld (Lauda-Königshofen, Germany).

### Preparation of the PEG, PVP, and SDS solutions

Accordingly, 0.5 wt.% of the PEG, PVP, and SDS solutions was prepared by dissolving 0.5 g of each powder to 99.5 g of DIw, followed by mixing. The polymers (i.e., PEG and PVP) were mixed through mild magnetic stirring at room temperature (23 ± 1 °C) without heating to avoid damage. These solutions were then left for more than 24 h to equilibrate. The PS microparticle suspension was centrifuged at 4,000 RPM for 5 min, and then suspended to each PEG solution. The PS concentration in the PEG solution was 0.1% (v/v).

### Preparation of the glass substrate and droplet deposition conditions

The droplet volume, which was controlled by a micropipette, was 1 ± 0.05 μl. The experimental conditions were 23 ± 1 °C (temperature) and 20 ± 3% (relative humidity). The glass substrates were cleaned by Piranha treatment in a laboratory fume hood. The slide glasses were soaked for 20 min in Piranha solution, which was a mixture of concentrated sulfuric acid (H_2_SO_4_) and 30% hydrogen peroxide (H_2_O_2_). The ratio of the two solutions was 4:1. The cleaned glasses were then rinsed with DIw and dried with nitrogen gas.

### Analyses of the deposition patterns and PEG-concentrations

First, we captured the background images before dropping the solutions. We then obtained the original images of the deposition patterns. The intensities of the background images were excluded from the original images to remove the light source effect. We subsequently analyzed the deposition patterns from these background excluded images (Fig. 1a–c) by plotting line A-A′ using the *ImageJ* software (National Institutes of Health). The inverted grayscale and the local particle density were found to be in a linear relationship^17^.

Second, the PEG concentration was carefully determined (Supplementary Fig. 1), as reported by Kajiya *et al*.^17^. We obtained the images of the fluorescent particles in a droplet using an inverted fluorescence microscope (IX71, Olympus Co., Japan). We then determined the intensity profile along the radial direction using the *ImageJ* software (Supplementary Fig. 1b). The lateral profile was obtained with a portable microscope (iCamscope, Sometech Co., Korea), which were located on the side of the droplet. Since the intensity increased almost linearly versus the lateral height, the initial intensity profile was normalized with the height profile of a droplet. Then, the intensity was calibrated with the initial bulk concentrations which was known concentration values. Finally, the calculated intensity was then converted to a concentration profile as shown in Supplementary Fig. 1e. The detailed description of determining polymer concentration of a droplet can be found elsewhere^17^.

As shown in Supplementary Fig. 2, we measured surface tension and viscosity of PEG solution at various concentrations, respectively. Then, we found each curve-fitting equation which can determine surface tension and viscosity with a given concentration, respectively. The profiles of surface tension and viscosity in a droplet were depicted as shown in Fig. 3b and c.

### Instruments

The surface tension was measured using a surface tension analyzer (SEO Co., Korea) at 23 ± 2 °C. The viscosities for the lowly and highly viscous liquids were measured at 25 °C using SV-10 (AND Co., Japan) and VISCO ELITE (Fungilab Co., Spain), respectively. The side view images were captured using a CMOS camera (iCamscope, Sometech Co., Korea). The top view images were observed through an inverted microscope (IX71 Olympus Co., Japan) at 40× and 400× magnifications. Lastly, a digital microscope camera (Jenoptik Co., Germany) was used to capture the images with the frame rate of 30 fps. The images recorded in a video on a frame-by-frame basis were examined to successfully track moving particles and determine their velocities and vortex lengths, respectively. Considering minimum resolution of the microscope-connected camera (0.5 μm), the uncertainty in measuring vortex length and flow velocities are less than 4.5%. Also, the uncertainty in property measurements is less than 1.0% and that for determining PEG concentration is less than 5%.

## Electronic supplementary material

Supplementary Figures

Comparison of Marangoni vortex with tracking the motion of the suspended particles near the contact line with respect to time

Comparison of Marangoni vortex with varying particle size in PEG and SDS
