# Supplementary material for: Altering the coffee-ring effect by adding a surfactant-like viscous polymer solution
Source: Sci Rep. 2017 Mar 29;7:500. doi: 10.1038/s41598-017-00497-x (PMC5428518; doi:10.1038/s41598-017-00497-x)
Supplement: Supplementary file 1 — Supplementary Figures [file 41598_2017_497_MOESM1_ESM.pdf]

Supplementary information

Altering the coffee-ring effect by adding a surfactant-like viscous polymer solution

Changdeok Seo+, Daeho Jang+, Jongjin Chae, Sehyun Shin*

School of Mechanical Engineering, Korea University, Seoul 136-701, Republic of Korea

*Corresponding author: Sehyun Shin, School of Mechanical Engineering, Korea University, Seoul 136-701, Republic of Korea; Tel.: +82 2 3290 3377; Fax: +82 2 928 5825; e-mail: lexerdshin@korea.ac.kr

+These two authors contributed equally to this work.

Supplementary Figure 1. Fluorescence-intensity-based concentration analysis method. (a) Captured image from fluorescence microscopy image. (b) Plotted intensity profiles along the line A to A′. (c) An image of side view of a droplet, (d) Height profile of the droplet, (e) Calculated concentration profiles at various times.

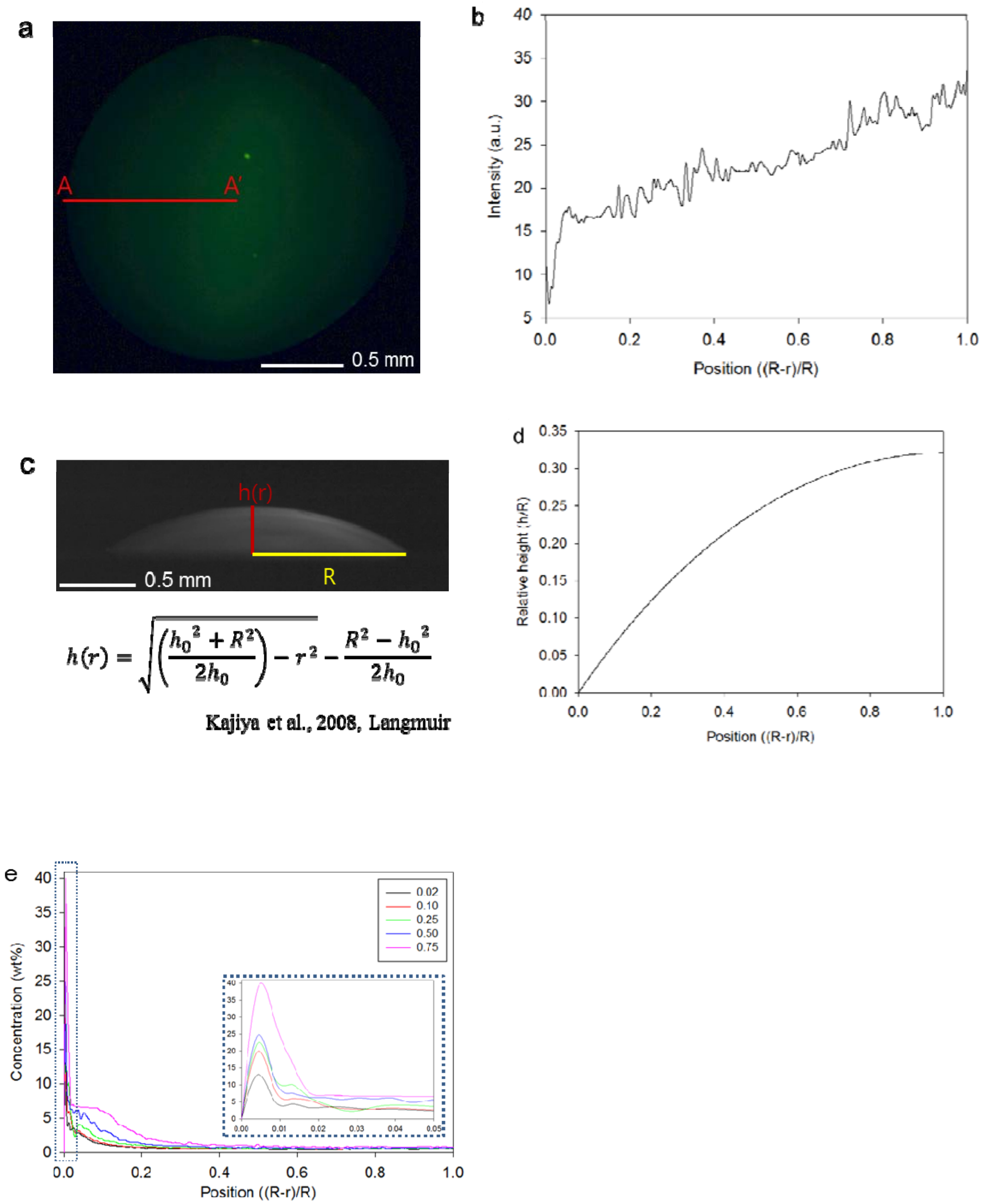

Supplementary Figure 2. Properties of the PEG solutions with varying concentration (a) surface tension, (b) viscosity.

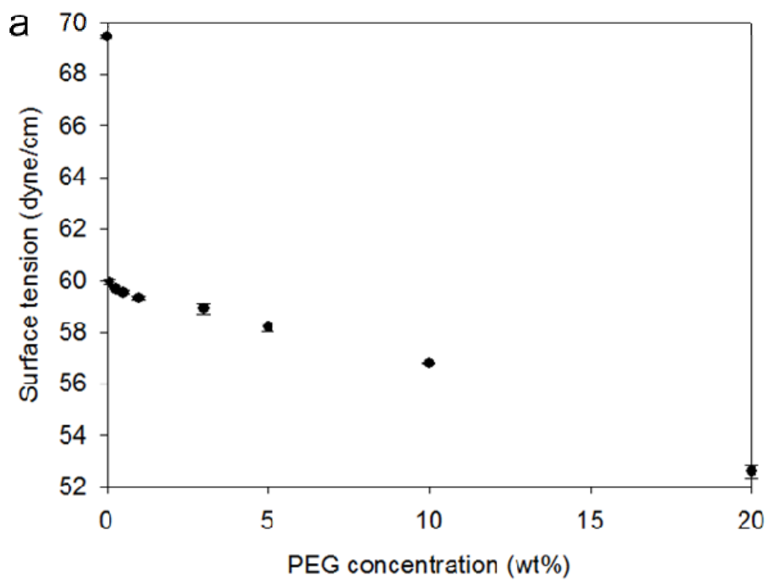

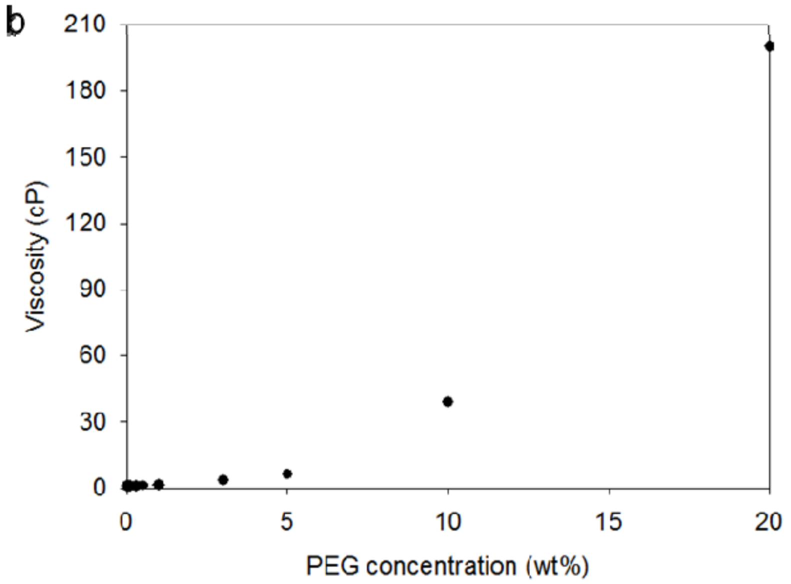

Supplementary Figure 3. Deposition patterns for (a) mixture of SDS and glycerol, (b) mixture of SDS and PVP.

a

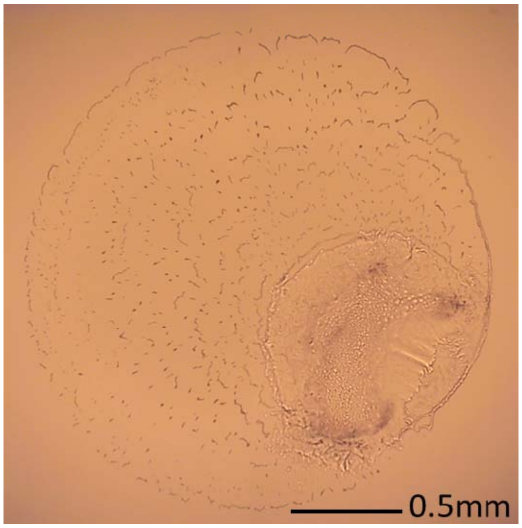

b

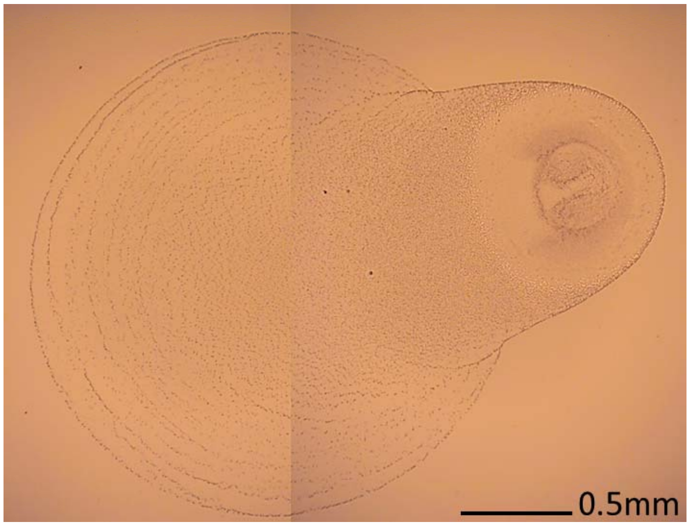

Supplementary Figure 4. Variation of droplet shape during evaporation for different PEG concentrations.

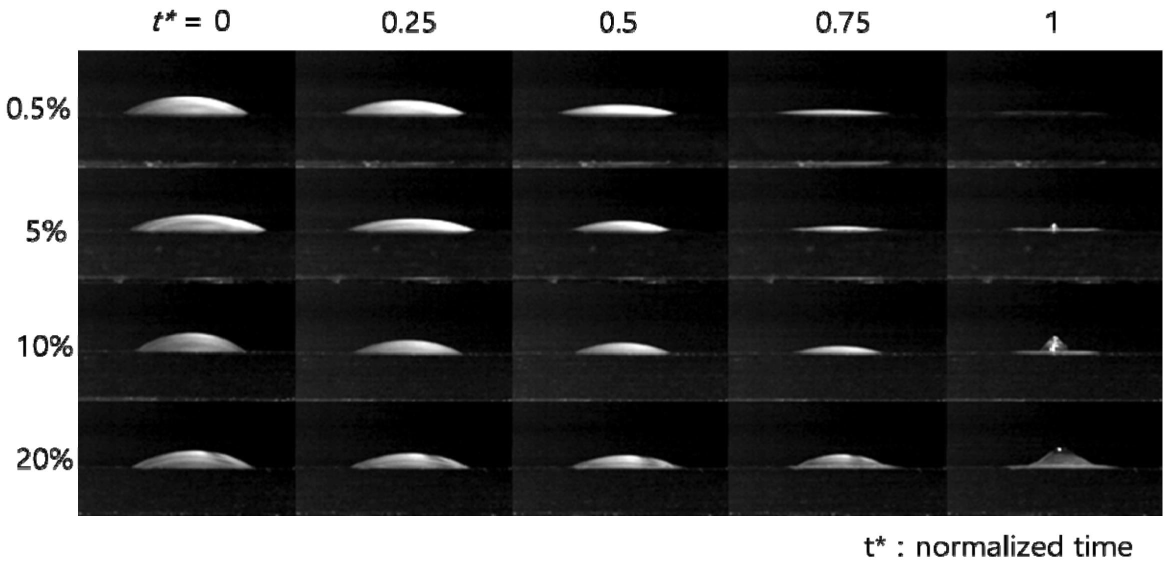

Video 1 Legends

Comparison of Marangoni vortex with tracking the motion of the suspended particles near the contact line with respect to time.

SDS resulted in strong and fast Marangoni vortex followed by unstable pinning-depinning process (upper window).

PEG resulted in stable Marangoni vortex and pinning-depinning process (lower window).

PEG droplet yields more frequent and denser rings than the SDS droplet.

PEG droplet formed the most uniform deposition pattern among the tested samples.

Video 2 Legends

Comparison of Marangoni vortex with varying particle size in PEG and SDS
